# Genetic Characterization of the M RNA Segment of Crimean Congo Hemorrhagic Fever Virus Strains, China

**DOI:** 10.3201/eid0801.010087

**Published:** 2002-01

**Authors:** Anna Papa, Benjiang Ma, Sophie Kouidou, Qing Tang, Changshou Hang, Antonis Antoniadis

**Affiliations:** * Aristotelian University of Thessaloniki, Thessaloniki, Greece †Chinese Academy of Preventive Medicine, Beijing, China

**Keywords:** *Nairovirus*, Crimean-Congo hemorrhagic fever, M RNA segment, China

## Abstract

We report the genetic characterization of the M RNA segment of *Crimean Congo hemorrhagic fever virus* (CCHFV). Two CCHFV strains isolated in Xinjiang Province, a region endemic for CCHF in northwestern China, were studied. These strains, designated BA66019 and BA8402, were isolated in 1965 and 1984 from a CCHF patient and *Hyalomma* ticks, respectively. Viral RNA was extracted from suckling mouse brains infected with these two strains, amplified, and sequenced. The full-length M RNA, consisting of 5.3 kb, was determined for both strains. The coding nucleotide sequences of the two strains differed from each other by 17.5% and from the reference CCHFV strain IbAr10200 by a mean of 22%, suggesting that the genus *Nairovirus* comprises a group of genetically highly diverse strains.

Crimean Congo hemorrhagic fever (CCHF), one of the most severe human viral diseases, has a death rate of up to 50%. CCHF is a public health problem in many regions of the world, including Africa, Middle East, southern and eastern Europe, and Western Asia (*1*–*9*). The causative agent, *Crimean Congo hemorrhagic fever virus* (CCHFV), is the type species of the genus *Nairovirus* in the *Bunyaviridae* family. The virus is transmitted to humans by the bite of *Ixodid* ticks (mostly of the *Hyalomma* genus) or by contact with blood or tissues from human patients or infected livestock. After an incubation period of 3 to 7 days, the patient has sudden onset of fever, chills, myalgia, headache, and rapidly evolving severe illness, followed by a hemorrhagic state with bleeding from the mucous membranes and petechiae, associated with thrombocytopenia and leukopenia (*10*). The highly pathogenic nature of the virus; the risk for spread from person to person through exposure to infected blood, respiratory secretions and excreta, resulting occasionally in serious nosocomial outbreaks; and the lack of an effective and safe therapy indicate the need for adequate guidelines for management of viral hemorrhagic fevers.

CCHFV, like all members of the genus, is a negative-stranded RNA virus with a tripartite genome consisting of a small (S), a medium (M), and a large (L) segment. The S RNA segment codes for the nucleocapsid (N) protein, and the M RNA segment codes for the glycoprotein precursor, resulting in the two envelope glycoproteins G1 and G2, while the L segment encodes the putative RNA-dependent polymerase. The first 8 to 13 nucleotide bases at the 3’ ends of all three RNA segments have a sequence that is conserved in the viruses of this genus, with a complementary consensus sequence occurring at the 5’ end; the ends of the segments are noncovalently linked so that the RNA occurs in a loosely bound circular configuration within the nucleocapsids (*11*). The M segment of nairoviruses is 30% to 50% larger than the M segments of members of other genera in the *Bunyavidae* family and has a potential coding capacity of up to 240 kDa of protein (*12*). CCHFV shares some common antigenic and genetic properties with *Dugbe virus* (DUGV), another member of the genus *Nairovirus*; some G1 epitopes may be conserved between the two viruses. Current knowledge of the molecular heterogeneity of CCHFV strains circulating in different parts of the world is very limited.

In China, the first CCHF cases were observed in 1965, when CCHFV strain BA66019 was isolated from a patient who lived in Xinjiang Province, an autonomous region in northwestern China which is the most CCHF-endemic area in the country. Another strain, BA8402, was isolated in 1984 from *Hyalomma asiaticum* ticks from the same region. The full-length S genome segment of these strains has been sequenced and analyzed (*13*). Over a 30-year period (1965 to 1994), 260 CCHF cases have been reported in China, 54 (21%) of them fatal. In 1997, an outbreak occurred in Honghai village (Bachu County, Xinjiang Province). During the 45-day outbreak period, 26 cases were reported, 5 of them fatal. Antibodies to CCHFV have been detected in humans and animals in the following provinces: Xinjiang, Qinghai, Sichuan, Yunnan, Anhui, Hennan, and Inner Mongolia (*13*).

Sequences of short S RNA CCHFV fragments from different parts of the world have shown considerable genetic differences (*14*–*16*). However, knowledge about the M RNA fragment is very limited, as no reports have been published and the only available complete M RNA sequence is that of the reference strain IbAr10200, isolated in 1970 from ticks in Nigeria (GenBank accession number U39455).

The M gene is critical for immunity and pathogenicity, as well as for vaccine development. To define the molecular variability among CCHFV strains, we determined the nucleotide sequences of the M RNA genome segment of two CCHFV strains (BA66019 and BA8402) isolated in China. We also compared their predicted amino acid sequences with the respective sequences of the reference strain.

## Virus Strains and Methods

Two CCHFV strains were studied. The first, strain BA66019, was isolated in 1965 from an ill resident of Xinjiang Province. The second strain, BA8402, was isolated in 1984 from *Hyalomma asiaticum* ticks from the same geographic region. Both strains were propagated in Vero E6 cells.

RNA was extracted from suckling mouse brains infected with the two strains by using Trizol reagent (Invitrogen Corp., Carlsbad, CA) according to the manufacturer’s instructions. Complementary DNA was prepared with one gene-specific primer and random hexamers. We used 12 sets of primers to amplify 12 overlapping fragments of the whole M RNA of the two CCHFV strains. Primers were designed with the OLIGO primer analysis software (Molecular Biology Insights, Cascade, CO), first based on the known sequences of CCHFV strain IbAr10200 and later on the basis of the sequences determined. The amplified polymerase chain reaction (PCR) products were purified and sequenced in an ABI 373A Perkin Elmer fluorescent dye automated sequencer (Perkin Elmer/Applied Biosystems, Foster City, CA). Sequences were aligned by ClustalW and genetic distances were estimated by the Kimura 2-parameter method with DNAdist program from the PHYLIP package (*17*). A phylogenetic tree was constructed by using Seqboot, DNAdist, Fitch and Consence from the same software package. Hydrophobicity plots were done by the Kyte and Doolittle method (*18*).

## Results

Twelve overlapping M segment PCR products were amplified and sequenced in both directions, covering a continuous region of the whole M RNA segment of two CCHFV isolates, BA66019 and BA8402; the M RNA sequences were submitted to GenBank and assigned accession numbers AF350448 and 350449, respectively. The overlapping sequences were identical.

**Figure 1:**
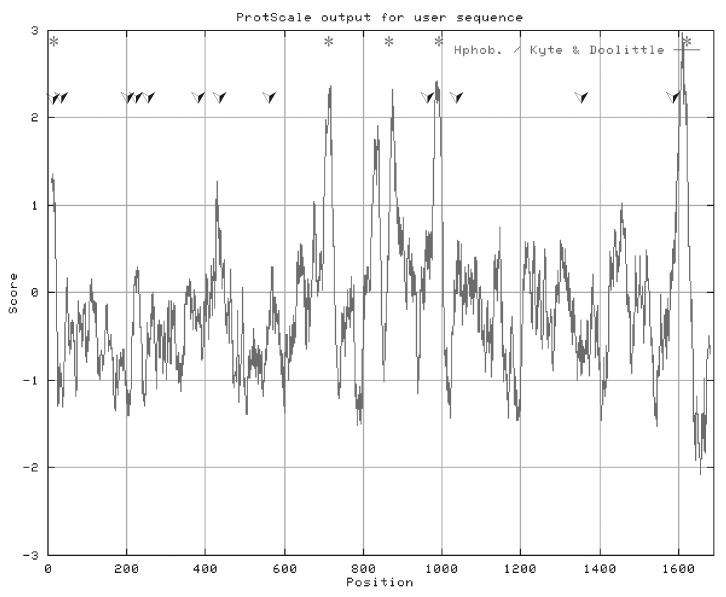
Hydropathy plot of the *Crimean Congo hemorrhagic fever virus* (CCHFV) strain BA66019 M segment open reading frame representing 1689 amino acids. Twelve potential N-linked glycosylation sites are indicated with an asterisk (*). The plot was constructed by the method of Kyte and Doolittle (1982) with a window size of 21. Hydrophobic residues appear above the line and hydrophilic residues below the line. Five strongly hydrophobic regions are marked (_).

The complete M RNA genome of BA66019 was found to be 5,368 nt long, with a nucleotide composition of 30.9% adenine, 24.7% uracile, 22.1% guanine, and 22.3% cytocine (G/C content 44.4%). The 5’ and 3’ ends are perfectly complementary for 13 nt and partially complementary for 24 nt, with a mismatch at positions 14 and 16. A long open reading frame (ORF) is observed from the first methionine (AUG) start codon at nucleotide positions 78-80 to a stop codon at 5,142-5,144. A second in-frame initiation codon is at nucleotide position 93-95, in the same position as the start codon of the 10,200 strain. The ORF could encode a polypeptide of 1,689 amino acids with a predicted molecular weight of 186,952.8. The polypeptide sequence contains 12 potential asparagine-linked glycosylation sites (Asp-X-Ser/Thr), and the theoretical pI is 7.99. A hydropathy plot of the M segment protein sequence shows at least six highly hydrophobic, potentially membrane-spanning regions (Figure 1).

The complete M RNA genome of BA8402 is 5367 nt long, with a nucleotide composition of 30.8% adenine, 25.2% uracile, 21.8% guanine, and 22.2% cytocine (G/C content 44.6%). The 5’ and 3’ ends are complementary and identical with the respective nucleotides of strain BA66019. A long ORF is observed from the first methionine start codon at 78-80 to a stop codon at nt 5,142-5,144. Similar to BA66019 strain, a second in-frame initiation codon is also seen at position 93-95. The ORF could encode a polypeptide of 1,689 amino acids, with a predicted molecular weight of 187,194.1. The polypeptide sequence contains 10 potential asparagine-linked glycosylation sites; the theoretical pI is 7.82. A hydropathy plot of the M segment protein sequence shows at least six highly hydrophobic, potentially membrane-spanning regions, similar to the plot of strain BA66019 (Figure 1).

Alignment of the M RNA sequences of the two Chinese CCHFV strains with that of the reference strain showed considerable variability among the three strains. Greater divergence was observed at the first 240 amino acids of the M genome. The nucleotide and amino acid differences between CCHFV strains in M RNA coding regions are as follows: BA66019 versus BA8402, 17.55% and 13.09%; BA66019 versus IbAr10200, 21.37% and 16.53%; and BA8402 versus IbAr10200, 22.57% and 17.04%, respectively.

**Figure 2:**
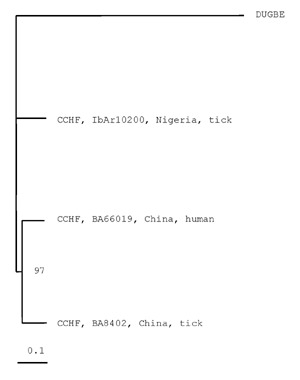
Phylogenetic tree based on 4,722 nt of the medium (M) RNA segment, including the two Chinese *Crimean Congo hemorrhagic fever virus* (CCHFV) strains BA66019 and BA8402 and the CCHFV strain IbAr10200, GenBank accession number U39455. *Dugbe virus* (DUGV) strain ArD44313, GenBank accession number M94133, was used as outgroup. Horizontal distances are proportional to nucleotide difference; vertical distances are for graphic display only. Bootstrap support (in %) is indicated at the respective branch.

Both Chinese strains have an extra proline (CCC for BA66019 and CCU for BA8402) at position 126, which is missing from strain IBAr10200. A phylogenetic tree was constructed; DUGV was used as outgroup (Figure 2).

## Conclusion

The full-length M RNA sequences from two CCHFV strains were determined, representing the first published genetic characterization of CCHFV M RNA segment. The two strains were isolated in the region of China most endemic for CCHF.

Phylogenetic analysis of S RNA sequences showed that at least three subtypes of CCHFV N protein are in circulation, with substantial genetic variability among them, as a divergence of approximately 20% was observed among the three major clusters. One cluster contains sequences from Iran and West Africa; a second cluster contains viruses from locations throughout Africa, Asia, and the Middle East; and a third cluster contains a single virus from Greece (strain AP92). In addition, no obvious correlation with geographic distribution or time of isolation was seen (*15*,*16*). We were unable to construct an informative phylogenetic tree with M RNA sequences, as there are only three complete CCHFV M RNA sequences. However, in the constructed tree, the two Chinese strains cluster together with strong bootstrap support (97%), differing from the Nigerian tick isolate. The considerable difference among the two Chinese strains and the IbAr10200 strain suggests that CCHF viruses comprise a genetically diverse group.

Prominent features of the M RNA segment are a high degree of divergence at the first part of the M genome, along with conservation of the middle and last regions and the 10-nt termini, which are conserved in all nairoviruses. In the S segment, a genetic difference of 3.3% was found between the Chinese BA66019 and BA8402 strains and a 15% difference between them and strain IbAr10200 (*13*). Estimation of the genetic distances of the M segment sequences after removal of the first 250 amino acids showed that the two Chinese strains differed from each other by 10% and from the IbAr10200 strain by 13%. The high degree of divergence of the first part of the genome (excluding the conserved termini) between the three strains indicates either that its function does not depend on a specific primary sequence, or, most probably, that the functional variability of these elements has no major impact on the CCHFV life cycle. Whether reassortment of RNA segments is a factor in CCHF epidemiology is not known.

The high degree of variability posed problems for the design of PCR and sequencing primers. First we designed primers based on the sequences of the reference strain, but new ones had to be designed on the basis of the Chinese sequences, as some differences in the genome led to annealing failure. Further M RNA sequences will help in determining the most conserved regions and subsequently the design of the most effective primers.

The nucleotide sequence and the coding strategy of the M RNA segment of DUGV have been determined (*19*). Comparison of the sequences of the three CCHFV and DUGV M segments showed that the M segment of DUGV is shorter than those of CCHFV. However, there is no evidence of truncation of the DUGV glycoprotein precursor, as observed at the N protein of DUGV. This N protein was found to be truncated relative to CCHFV and Hazara virus N proteins. This truncation may be a recent event in evolutionary terms, involving the mutation of an amino acid codon to a UGA stop codon (*20*). In estimating our results, we had in mind the first initiation codon, which is thought to have more favorable flanking sequences for initiation of protein synthesis, although use of leaky scanning mechanism could be the reason of initiation at the second AUG codon (*21*).

By analogy to other viruses of the *Bunyaviridae* family, the 1,689-amino acid product is supposed to be the precursor of the two glycoproteins, G1 and G2. Structural features may play a role in immunologic recognition of most important epitopes on the G1 and G2 proteins of these viruses. The positions of all 79 cysteine residues in both G1 and G2 proteins are conserved, suggesting that these two proteins are structurally similar. In addition, we observed that the glycosylation sites are conserved.

In conclusion, we determined the complete M RNA sequences of two CCHFV strains isolated in China. Additional complete genome sequences from human and tick CCHFV strains isolated in different parts of the world will help in identifying important conserved regions for the application of successful diagnostic methods, for the design of effective PCR and sequencing primers, and for the design of an effective vaccine. Analysis of such sequences will also elucidate the epidemiology of the virus, the exact phylogenetic relationship among different CCHFV strains, and subsequently the genetic evolution of the virus.
